# Antiproliferative and Antiangiogenic Effects of *Punica granatum* Juice (PGJ) in Multiple Myeloma (MM)

**DOI:** 10.3390/nu8100611

**Published:** 2016-10-01

**Authors:** Daniele Tibullo, Nunzia Caporarello, Cesarina Giallongo, Carmelina Daniela Anfuso, Claudia Genovese, Carmen Arlotta, Fabrizio Puglisi, Nunziatina L. Parrinello, Vincenzo Bramanti, Alessandra Romano, Gabriella Lupo, Valeria Toscano, Roberto Avola, Maria Violetta Brundo, Francesco Di Raimondo, Salvatore Antonio Raccuia

**Affiliations:** 1 Section of Hematology, Department of Surgery and Medical Specialties, University of Catania, Catania 95125, Italy; d.tibullo@unict.it (D.T.); cesarinagiallongo@yahoo.it (C.G.); puglisi.fabri@gmail.com (F.P.); lauraparrinello@tiscali.it (N.L.P.); alessandraromano@google.it (A.R.); diraimon@unict.it (F.D.R.); 2 Department of Biological, Geological and Environmental Sciences, University of Catania, Catania 95125, Italy; claudia.genovese@cnr.it (C.G.); carmen.arlotta@isafom.cnr.it (C.A.); salvatore.raccuia@cnr.it (S.A.R.); 3 Department of Biomedical and Biotechnological Sciences, University of Catania, Catania 95125, Italy; nunzia.caporarello@gmail.com (N.C.); anfudan@unict.it (C.D.A.); V.bramanti@unict.it (V.B.); lupogab@unict.it (G.L.); alessandraromano@google.it (R.A.); 4 Institute for Agricultural and Forest Systems in the Mediterranean, National Research Council, Catania 95125, Italy; valeria.toscano@cnr.it

**Keywords:** *Punica granatum* juice, multiple myeloma, proliferation, angiogenesis

## Abstract

Multiple myeloma (MM) is a clonal B-cell malignancy characterized by an accumulation of clonal plasma cells (PC) in the bone marrow (BM) leading to bone destruction and BM failure. Despite recent advances in pharmacological therapy, MM remains a largely incurable pathology. Therefore, novel effective and less toxic agents are urgently necessary. In the last few years, pomegranate has been studied for its potential therapeutic properties including treatment and prevention of cancer. Pomegranate juice (PGJ) contains a number of potential active compounds including organic acids, vitamins, sugars, and phenolic components that are all responsible of the pro-apoptotic effects observed in tumor cell line. The aim of present investigation is to assess the antiproliferative and antiangiogenic potential of the PGJ in human multiple myeloma cell lines. Our data demonstrate the anti-proliferative potential of PGJ in MM cells; its ability to induce G0/G1 cell cycle block and its anti-angiogenic effects. Interestingly, sequential combination of bortezomib/PGJ improved the cytotoxic effect of the proteosome inhibitor. We investigated the effect of PGJ on angiogenesis and cell migration/invasion. Interestingly, we observed an inhibitory effect on the tube formation, microvessel outgrowth aorting ring and decreased cell migration and invasion as showed by wound-healing and transwell assays, respectively. Analysis of angiogenic genes expression in endothelial cells confirmed the anti-angiogenic properties of pomegranate. Therefore, PGJ administration could represent a good tool in order to identify novel therapeutic strategies for MM treatment, exploiting its anti-proliferative and anti-angiogenic effects. Finally, the present research supports the evidence that PGJ could play a key role of a future therapeutic approach for treatment of MM in order to optimize the pharmacological effect of bortezomib, especially as adjuvant after treatment.

## 1. Introduction

Multiple myeloma (MM) is a clonal B-cell malignancy characterized by accumulation of clonal plasma cells (PC) in the bone marrow (BM) leading to bone destruction and BM failure [1,2,3]. MM encompasses a spectrum of clinical variants ranging from benign Monoclonal Gammopathies of Undetermined Significance (MGUS) and smoldering/indolent MM, to more aggressive, disseminated forms of MM and plasma cell leukemia. Despite recent advances in proteasome inhibitor and immunomodulatory drug-based therapies [4,5], and MM remains largely incurable [2,6]. The genetic complexity of myeloma is due to intraclonal heterogeneity in the myeloma-propagating cell [7,8]. Multiple mutations in different pathways trigger a deregulation of the intrinsic biology of the PC, leading to the features of myeloma [7,8,9]. The sequential acquisition of multiple genetic events can lead to disease progression and the development of treatment-resistant disease [8,9]. Therefore, novel effective and less toxic agents are urgently necessary in order to treat patients affected by multiple myeloma. The development of novel therapeutic compounds without significant adverse side effects is considered an important area for immunopharmacological studies. A wide variety of natural compounds possesses significant cytotoxic as well as chemopreventive activity, through induction of cancer cell apoptosis [10]. Over 60% of currently used anticancer agents are derived from natural sources, including plants, marine organisms and microorganisms.

*Punica granatum* L., also known as pomegranate, belonging to the Punicaceae family, has been gaining popularity as a nutraceutical food having potential beneficial effects on health, including prevention and/or treatment of oncologic diseases, cardiovascular and neurological disorders, metabolic diseases [11,12]. Moreover, pomegranate has been studied for its potential therapeutic properties including treatment and prevention of cancer [13,14]. Pomegranate fruit is widely consumed fresh as well as in processed forms, such as juice, jams, sauce, and wine. The pharmacological effects of PGJ are related to a large number of phytochemicals, including hydrolyzable tannins and related compounds (ellagitanin, punicalagin, pedunculagin, punicalin, gallagic acid, ellagic acid and gallic acid), flavonoids (anthocyanins and catechins), flavonols (quercetin and kaempferol), flavones (apigenin and luteolin), and conjugated fatty acids (punicic acid), present in discrete anatomical parts, such as peel (pericarp or husk), juice, and seeds [13,15]. PGJ, extracts, and phytoconstituents have been extensively studied preclinically for their anticarcinogenic and cancer chemopreventive effects in colon, lung, skin, and prostate cancer [16]. 

Based on numerous in vitro studies, several pomegranate products and phytoconstituents exhibited cytotoxic, antiproliferative, proapoptotic, antiangiogenic, antiinvasive, and antimetastatic effects against estrogen receptor-positive and -negative breast carcinoma cells [17,18,19,20,21,22,23,24,25].

Pomegranate seed oil and fermented juice concentrate suppressed 7,12-dimethyl benz(*a*)anthracene (DMBA)-induced precancerous mammary gland lesions ex vivo [26] and pomegranate extract inhibited the growth of xenografted BT-474 tumors in vivo [23].

Recently, some authors reported for the first time that a pomegranate formulation (emulsion) containing the most bioactive phytochemicals present in the whole fruit affords a remarkable chemopreventive effect against DMBA-induced mammary tumorigenesis in rats [27].

The aim of the present investigation is to assess the antiproliferative and antiangiogenic potential of PGJ in human multiple myeloma cell lines.

## 2. Experimental Section

### 2.1. Reagents and Compounds

Recombinant human VEGF-A (VEGF-A_165_ isoform) was obtained from Peprotech (Rocky Hill, NJ, USA). The serum-free endothelial cell basal medium (EBM) was obtained from ScienceCell Research Laboratories (San Diego, CA, USA). The proteosome inhibitor bortezomib (BTZ) was used at 30 nM (Takeda, Italy).

### 2.2. Pomegranate Juice

Pomegranate juice (PGJ) was extracted by plants of five-year-old Wonderful varieties. The plants were produced under organic agricultural practices in an experimental farmland of the section of National Research Council—Institute for Agricultural and Forest Systems in the Mediterranean (ISAFOM).

Pomegranate fruits were harvested at physiological maturity in October 2014 and randomly collected from each of four geographical orientation of tree. The fruits were stored at 4 °C for three days. After manual separations of the arils, the PGJ was obtained by mechanical press. Subsequently, the PGJ was centrifuged at 5000 rpm for 5 min at room temperature and then filtered by sterile syringe with filters 0.22 µm (Invitrogen, Paisley, UK). Afterwards, each aliquot (20 mL) of the juice was collected into specific vials and stored at −20 °C.

### 2.3. Phenolic Compounds by HPLC Analysis

After being thawed at room temperature, the sample of the PGJ was analyzed using a liquid chromatography system Dionex UltiMate 3000 (Thermo Fisher Scientific, Waltham, MA, USA), including a solvent rack, a quaternary pump with an integrated four-channel degasser, a thermostatted column compartment, an autosampler and a four wavelength UV-Vis detector. Separation was performed on a column Dionex Acclaim 120, C18, 3 µm (4.6 × 150 mm) (Thermo Fisher Scientific, Waltham, MA, USA) with a gradient program at a flow rate of 0.8 mL·min^−1^. Column temperature was maintained at 30 °C and the injection volume was 20 µL. The mobile phases consisted of water and formic acid (95:5, v/v) (eluent A) and methanol (eluent B). The gradient started with 1% B to reach 20% B at 20 min, 40% B at 30 min, 95% B at 35 min and 1% B after 41 min. The chromatograms were recorded simultaneously at 280, 360 and 520 nm. Calculation of concentrations was based on the external standard method. The HPLC analysis was replicated three times and the average values are reported in Table 1.

### 2.4. Cell Cultures and Cell Viability Assay

MM cell lines KMS26, MM1S and U266 (established from peripheral blood of a multiple myeloma patients in refractory and terminal stage) were cultured in suspension using RPMI1640 medium supplemented with 10% or 20% fetal bovine serum (FBS) and 1% penicillin/streptomycin at 37 °C and 5% CO_2_. Human lymphocytes and monocytes were isolated, after informed consent, from fresh buffy coat of healthy volunteers provided by the Transfusional Centre of E. Muscatello Hospital—Augusta (SR). Monocytes and lymphocytes then were purified from the lymphomonocytic population by positive isolation using magnetic beads coated with goat anti-mouse CD14+ IgG and anti-mouse CD3+ IgG respectively (MiltenyiBiotec GmbH, Bergisch Gladbach, Germany) [28].

Human Brain Microvascular Endothelial Cells (HBMEC) (Innoprot, Elexalde Derio, Spain) were grown in monolayer in EBM supplemented with 5% fetal bovine serum (FBS), 1% endothelial cell growth supplement (ECGS), 100 U/mL penicillin and 100 mg/mL streptomycin. In studies involving serum-starvation, serum was reduced at 1% v/v.

Cell viability was assessed using ATPlite 1step assay (PerkinElmer, Milan, Italy) according to the manufacturers’ protocol. ATPlite TM 1step is an Adenosine TriPhosphate (ATP) monitoring system based on firefly (Photinus pyralis) luciferase. ATP is a marker for cell viability because it is present in all metabolically active cells. Because ATP concentration declines rapidly when cells undergo necrosis or apoptosis, monitoring ATP is a good indicator of proliferation effects [29,30,31]. 

This analysis is a patented luminescence test that analyzes cell proliferation and cytotoxicity in mammalian cells considering the light release induced by ATP-luciferase-d-luciferin reaction.

This luminescence assay is an alternative to colorimetric, fluorometric and radioisotopic assays for the quantitative evaluation of proliferation and cytotoxicity of cultured mammalian cells. ATP monitoring can be used to assess the cytocidal, cytostatic and proliferative effects of a wide range of drugs, biological response modifiers and biological compounds.

ATPlite 1 step (PerkinElmer, Waltham, Massachusetts MA, USA) is a true homogeneous high sensitivity ATP monitoring 1-step addition assay kit for the quantification of viable cells. Because the kit needs no stabilization of the luminescence signal, high throughput is preserved. Briefly, the 96-well black culture plate was taken from the incubator and equilibrated at room temperature for 30 min. Subsequently, to each well containing 100 μL of the cell suspension (5 × 105 cells/mL), 100 μL of reconstituted reagent was added and the plate was shaken for 20 min at 700 rpm using orbital shaker (Stuart Scienti c, Staffordshire, UK). The luminescence was measured using Victor3 (PerkinElmer, Milan, Italy). Viability of the cells was expressed as percentage of vitality of untreated cells.

MTT assay: To quantify cell viability in endothelial cells, the 3-[4,5-dimethylthiazol-2-yl]-2,5-diphenyl tetrasodium bromide (MTT) assay was used (Chemicon, Temecula, CA, USA). 10,000 cells/well were plated in 96-well plates and grown in complete medium, in the absence or in the presence of PGJ (3%, 6% or 12% v/v) for 24–48 or 72 h. At the end of treatment, the cells were incubated with MTT for 4 h; then, 100 μL dimethyl sulfoxide was added and the absorbance was read at 590 nm, as previously described [32].

### 2.5. Cell Cycle Analysis

Cells were washed and resuspended in cold 80% ethanol to obtain a final concentration of 0.5 × 10^6^ cells/mL for 1 h at 4 °C. The ethanol-fixed cells were centrifuged to remove ethanol and the pellet was resuspended in propidium iodide-staining reagent (0.1% Triton X-100), 0.1 mm Ethylenediaminetetraacetic acid (EDTA), 0.05 mg/mL RNase A and 50 mg/mL propidium iodide). Cells were stored in the dark at room temperature for about 3 h. Cells were then analyzed with a flow cytometer (FC500 Beckman Coulter; Beckman Coulter S.p.A., Milano, Italy) and the data were processed by the ModFit program (Verity Software House, version 4.0, Topsham ME 04086, US).

### 2.6. Cell Invasion Assay

In order to perform cell invasion/migration experiments, harvested cells (1 × 10^6^ cells/mL) were seeded into 8.0-μm-pore transwell inserts (Corning Life Sciences, Lowell, MA, USA) coated with Matrigel in the absence (control cells) or in the presence of PGJ (3% or 6% v/v) in medium containing 1% FBS. Cells migration was stimulated by addition of VEGF (50 ng/mL) to the lower well of the Boyden chamber. After 24 h, the cells were fixed with 95% ethanol and the invading cells were stained with 1× crystal violet. Invaded cells were stained with 0.1% crystal violet and counted after scanning membranes (×100 total magnification) by using ImageJ software (ImageJ 1.50e, National Institutes of Health, NIH, Bethesda, MD, USA). The membranes were also dissolved in acetic acid the density of invading cells was measured by reading the absorbance at 590 nm using a plate reader (Synergy 2-BioTek, Winooski, VT, USA).

### 2.7. Tube Formation Assay

The ability of cells to migrate and organize into capillary-like structures was carried out by using Matrigel (BD, Franklin Lakes, NJ, USA). Briefly, the cells were shifted to medium containing 1% serum for the 24 h. Next, the cells at 1 × 10^4^ were suspended in 200 µL of the same medium containing either PGJ at different concentrations or PGJ plus VEGF (50 ng/mL). The mixtures were seeded in 96-well plates covered with polymerized growth factor-reduced Matrigel matrix (BD, Franklin Lakes, NJ, USA), incubated for 4 h (37 °C, 5% CO_2_) and photographed at 100× magnification using an inverted Leica DM IRB microscope (Leica, Buffalo Grove, IL, USA) equipped with a Charge-Coupled Device (CCD) camera as previously described [33].

### 2.8. Aortic Ring Assay

Aortic rings were obtained by cross-sectioning the thoracic aorta of New Zealand white male rabbits at 1-mm intervals. Rings were placed individually on the bottom of 24-well plates, pre-coated with 150 μL of Matrigel. After 10 min, wells were rinsed with 150 μL of endothelial cell basal medium and incubated with the same medium containing 1% FBS in the absence or in the presence of VEGF-A (50 ng/mL) and the compound under test. The medium was changed in control and treated cells three times a week starting from day two. Aortic rings were observed daily for signs of angiogenic sprouting. The angiogenic response was measured by counting the length of neovessels sprouting out of the rings after 14 days. PGJ was renewed every two days during the assay.

### 2.9. Wound Healing Assay

Twenty-four well tissue culture plates were seeded with HBMEC to a final density of 150,000 cells per well, and these were maintained at 37 °C and 5% CO_2_. Cells were cultured in 1% serum medium supplemented or not with 3% or 6% PGJ, or with PGJ added to VEGF-A (50 ng/mL)-stimulated cells. As described before, VEGF-A alone was used as a positive control, and 1% serum medium was a negative control. Wound closure was monitored within 24 h to 48 h of seeding. Confluent HBMEC monolayer was scratched with a p200 pipet tip. The wounds were photographed at 40× using phase-contrast microscope. After 24 h and 48 h, endothelial cells invading the wound were quantified by computerized analysis of the digitalized images. For each image acquired, areas of scratch were measured using ImageJ tools. The migration of cells toward the wounds was expressed as percentage of wound closure, as previously described [34].

### 2.10. Gene Expression Analysis

RNA was extracted by Trizol reagent (Invitrogen, Carlsbad, CA, USA). First strand cDNA was then synthesized with Applied Biosystem (Foster City, CA, USA) reverse transcription reagent [29]. PPARγ mRNA expression was assessed by TaqMan Gene Expression, Applied Biosystem and quantified using a fluorescence-based real-time detection method by 7900HT Fast Real Time PCR System (Life Technologies, Carlsbad, CA, USA). For each sample, the relative expression level of PPARγ (Hs01115514_m1) mRNA was normalized using Glyceraldehyde 3-phosphate dehydrogenase (GAPDH) (Hs02758991_g1) as an invariant control [35]. Analysis of angiogenic genes was performed using TaqMan Low Density Array Human Angiogenic Panel (Life Technologies, Carlsbad, CA, USA) that contains assays for 93 human genes in addition to three endogenous controls (18S, ACTB, GAPDH).

### 2.11. Statistical Analysis

Statistical significance between two groups was analyzed by Student’s *t*-test. One-way and two-way analysis of variance (ANOVA), followed by Tukey’s post hoc test, was used for multiple comparisons. *p* values < 0.05 were considered statistically significant.

## 3. Results

### 3.1. Effect of Pomegranate Juice on Multiple Myeloma Cell Viability

**Figure 1:**
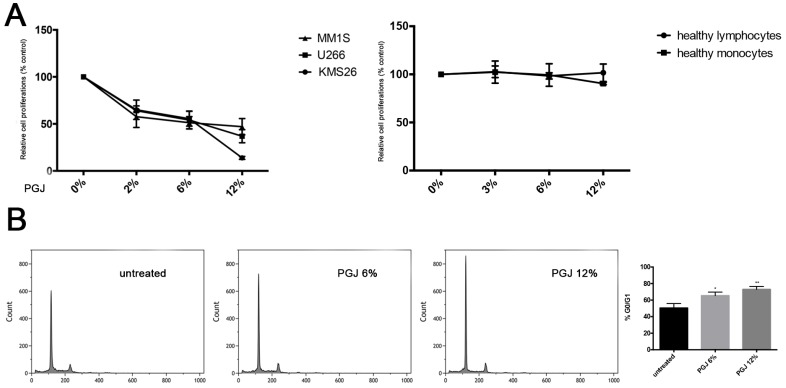

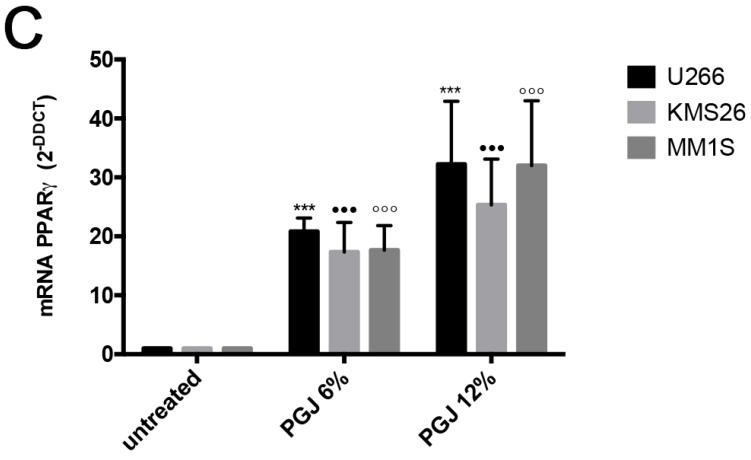
(**A**) the survival assay in U266, KMS26 and MM1S cell lines treated with PGJ. *Bars* represent the mean ± SEM of four independent experiments. ***/•••/◦◦◦ *p* < 0.0001 versus untreated cells; (**B**) the effect of PGJ treatment on G0/G1 phase in U266 cells. Cell cycle analysis was performed by the ModFit program ((Verity Software House, version 4.0, Topsham ME 04086, US). Results represent three independent experiments in triplicate (*p* < 0.002); (**C**) mRNA expression of PPARγ in MM cells treated with PGJ. *Bars* represent the mean ± SEM of four independent experiments. *** (U266 cells), ••• (KMS26 cells), ◦◦◦ (MM1S cells) *p* < 0.0001 versus untreated cells. (Calculated value of 2^−ΔΔCt^ in U266, KMS26, MM1S untreated was 1).

The anticancer activity of the PGJ was tested in U266, KMS26 and MM1S cell lines at different concentrations (3%, 6% and 12% of juice). After 24 h of treatment, we observed the inhibition of cell proliferation in a dose-dependent manner (*p* < 0.0001) (Figure 1A). The KMS26 cells showed a major sensitivity to the 12% PGJ exposure with 20% of proliferation only (*p* < 0.001). As showed in Figure 1B for U266 cell line, 6% and 12% PGJ induced G0/G1 cell cycle arrest in a dose-dependent manner (untreated: 50.58% ± 7%; 6% PGJ: 65.8% ± 5.2%; 12% PGJ: 73% ± 3%; *p* < 0.05), with a decrease of the percentage of cells in G(2)/M and S phase. In healthy lymphocytes and monocytes, the PGJ treatment did not show any effects (Figure 1A).

### 3.2. Pomegranate Juice Induces PPARγ Expression in Multiple Myeloma Cells

PGJ polyphenols can regulate the activation of PPARγ in several cancer cells [36]. It has been reported that this protein induces inhibition of cell proliferation in MM [37]. We observed a significant increase of PPARγ mRNA expression in U266 cells after treatment con PGJ in a dose-dependent manner. PGJ induced up-regulation of PPARγ of about 20 and 30 fold, respectively, at 6% and 12% (*p* < 0.0001) (Figure 1C), suggesting that this molecular mechanism may contribute to a PGJ anti-proliferative effect on MM cells.

### 3.3. Pomegranate Juice Inhibits Angiogenesis

**Figure 2:**
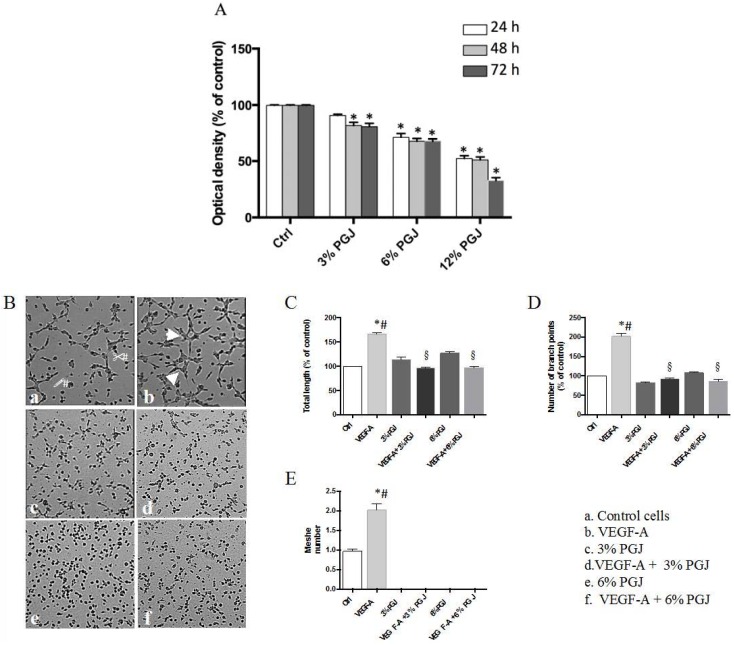
(**A**) Effect of PGJ on HBMEC viability. Cells (1 × 10^4^ cells/well) were cultured in complete medium in the absence or in the presence of PGJ at 3% or 6% or 12% (v/v). Cell viability was assessed by MTT assay. Values are expressed as mean ± SD of three independent experiments, each involving six different wells per condition. (* *p* < 0.05 vs. respective control); (**B**) Effect of PGJ on VEGF-A-induced HBMEC in vitro angiogenesis. Tube formation was evaluated with light microscopy and representative fields are shown. Panel (**a**): Control cells; panel (**b**): VEGF-A stimulated cells; panels (**c**) and (**e**): Cells treated with 3% and 6% PGJ, respectively; panels (**d**) and (**f**): Cells treated with 3% and 6% PGJ, respectively, in the presence of VEGF-A; Quantitative analysis of tube formation was indicated as tube length (**C**) and number of branch points (**D**) expressed as percentage of control cells. Image analysis of the total length and the number of branch points in the whole photographed area (representing central 70% of the well) were carried out by using Angiogenesis Analyzer tool for ImageJ (ImageJ 1.50e, National Institutes of Health, NIH, Bethesda, MD, USA). Values are expressed as a mean ± SD of three independent experiments performed in duplicate. Statistically significant differences by one-way analysis of variance (ANOVA) followed by Tukey’s test (*p* < 0.05) are indicated: (*) VEGF-A-stimulated cells vs. control; (§) 3% and 6% PGJ plus VEGF-A-treated cells vs. VEGF-A-stimulated cells.

**Figure 3:**
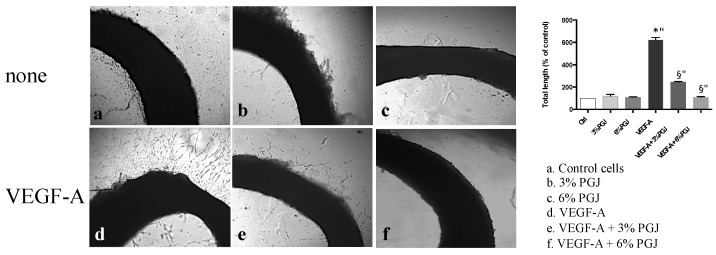
Developing microvessels from the intimal/subintimal layers of the aortic wall. Rabbit thoracic aortic rings were isolated and embedded on Matrigel, in the absence of VEGF-A ((**a**): untreated; (**b**): 3% PGJ; (**c**): 6% PGJ) or in the presence of 50 ng/ml VEGF-A ((**d**): VEGF-A alone; (**e**): 3% PGJ; (**f**): 6% PGJ). After 14 days, the angiogenic response was measured by counting the lenght of neovessels sprouting out of the rings. Representative photographs from a single experiment that was performed three times are shown. Statistically significant differences by one-way ANOVA followed by Tukey’s test (*p* < 0.05) are indicated: (*) VEGF-A-stimulated cells vs. control; (§) 3% and 6% PGJ plus VEGF-A-treated cells vs. VEGF-A-stimulated cells.

Since angiogenesis is associated with progression of MM [38], we first examined the effect of PGJ on HBMEC cell viability. As shown in Figure 2A, PGJ inhibited cell proliferation in a dose- and time-dependent manner by 18% and 19% at 48 h and 72 h, respectively, in the presence of 3% PGJ. Moreover, inhibitions by 29%, 31% and 32% at 24 h, 48 h, and 72 h, respectively, in the presence of 6% PGJ, were found. Since PGJ significantly reduced cell viability at 12%, this dose was not used for the subsequent tests. The direct effect of PGJ on angiogenesis was tested by using the tube formation and ex vivo rabbit aortic ring assays. We studied the effect of VEGF-A alone or in combination with 3% or 6% PGJ, on tubule formation of HBMEC by using the Matrigel assay. The results in Figure 2B show that the tubular networks formed on Matrigel vary in the different environments. The number of the interconnections between the tubes provides information on the way the HBMEC organize them and grow. In panel a (control), some cells are joined by projections or direct cell contact. Cellular projections that do not result in contact with other cells are also visible (arrows). In the presence of VEGF-A (panel b) the cells show sprouts which connect with similar projections originating from other cells to form a cell–cell contact. Some cells forming polygon structures are also visible (arrowheads). In the presence of 3% (panel c) or 6% (panel e) PGJ, the cells show few sprouts. Both 3% and 6% PGJ suppressed the formation of tube-like structures in presence of VEGF-A (panels d and f). As expected, the VEGF-A treatment significantly induced an enhancement of both tube length and number of branch points by 1.7 and 2.0 fold, respectively, in comparison to control unstimulated cells (Figure 2C,D). The total length and the number of branch points were significantly reduced by both concentrations of PGJ respect to VEGF-A-treated cells (*p* < 0.0001). The incubation of the cells with 3% or 6% PGJ in the presence of VEGF-A significantly reduced tube total length by 1.7 fold after both treatments in comparison to VEGF-A-treated cells. Moreover, fewer branch points between cells incubated with 3% or 6% PGJ plus VEGF-A was observed, with a reduction by 2.2- and 2.3-fold, respectively, in comparison to growth factor stimulated cells. Moreover, PGJ in the presence or not of VEGF-A totally inhibited the formation of enclosed spaces in Matrigel (mesh number, panel e). The ex vivo rabbit aortic ring assay (Figure 3) showed an evident new vessel sprouting of endothelial tubes from aorta rings when the incubations were carried out in presence of VEGF (panel d). Total length was about 6 fold greater with VEGF-A than controls cells (panel a) or 3% and 6% PGJ treated cells (panels b and c). Panels e and f show the effects of both 3% and 6% PGJ, respectively, on VEGF-treated cells. As shown in the bar graph inset, there is a dose-dependent effect, with a higher concentration of PGJ leading to a greater reduction of outgrowth of vascular shoots.

### 3.4. Effect of Pomegranate Juice on Cell Invasion and Migration

**Figure 4:**
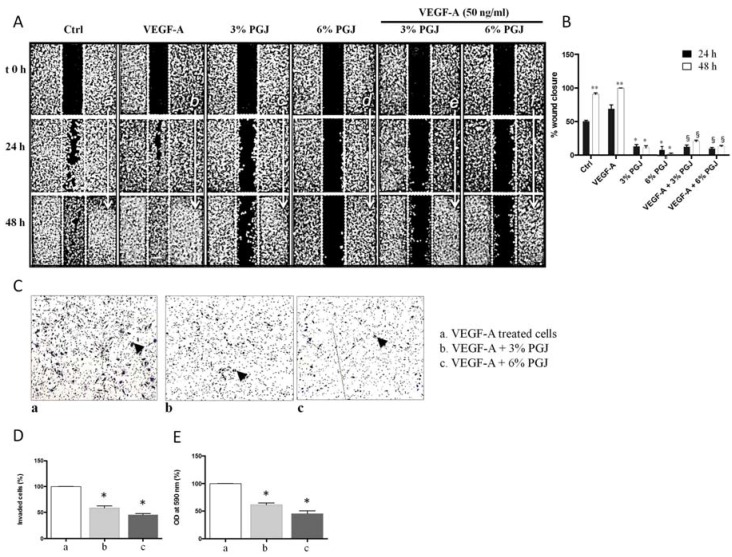
(**A’’**) Effect of PGJ on VEGF induced HBMEC migration (wound healing assay). Images of scratch photographed with at ×40 using phase-contrast microscope at different time point, 0, 24 and 48 h. (**a**) Control cells, 1% serum; (**b**) VEGF-A + 1% serum; (**c**) 3% PGJ; (**d**) 6% PGJ; (**e**) VEGF-A + 3% PGJ; (**f**) VEGF-A + 6% PGJ; (**B’’**) migration of HBMEC cells after wounding evaluated as percentage of wound closure respect to 48 h VEGF-A treated cells considered as 100% wound closure. The results are expressed as mean ± standard deviation. Statistically significant differences by one-way ANOVA followed by Tukey’s test (*p* < 0.05) are indicated: (#) non stimulated cells vs. control at 24 h; (*) 3% and 6% PGJ vs. control at 24- and 48 h; (**) VEGF-A-stimulated cells vs. respective control at at 24- and 48 h; (§) 3% and 6% PGJ plus VEGF-A-treated cells vs. VEGF-A-stimulated cells at 24- and 48 h; (**C’’**) effect of PGJ on VEGF-A induced HBMEC invasion. Harvested HBMEC (1 × 10^6^ cells/mL) were allowed to migrate through transwell membranes towards 50 ng/mL VEGF in the absence or in the presence of PGJ for 24 h. Cells that had migrated to the underside of the transwell membrane were fixed and evaluated with light microscopy. Representative fields are shown at 100× magnification; (**D’’**) average number (displayed as percentage of control) of HREC migrated in three different wells in each condition (*n* = 5 different fields of the same membrane); and (**E’’**) quantitative analysis of invaded cells, which were eluted using 10% acetic acid and measured optical density value at 590 nm. Data are the mean ± SD of three independent experiments. Statistically significant differences by one-way ANOVA followed by Tukey’s test (*p* < 0.05) are indicated: (*) 3% and 6% PGJ plus VEGF-A-treated cells vs. VEGF-A-stimulated cells.

Since endothelial cell migration is essential for the formation of new blood vessels during neo-angiogenesis, we evaluated the effects of PGJ treatment on the migratory and invasive properties of endothelial cells by using, respectively, the wound-healing and the transwell assays (Figure 4). As shown in panel A, the presence of VEGF-A in the medium increased HBMEC migration in comparison to control cells. Quantitative analysis showed in panel b revealed a VEGF-dictated increase of cell migration by almost 1.7- and 2.0-fold after 24 h and 48 h, respectively, compared with control cells. The incubation with VEGF-A plus 3% and 6% PGJ reduced the cell migration (panel a); this reduction was estimated at almost 6.0 fold when both 3% or 6% PGJ, respectively, were added to VEGF-A-treated cells for both 24 h and 48 h.

The effect of PGJ on HBMEC migration was also assessed by using transwell migration assays (panels c, d and e). A reduction of the number of invaded cells by 30% and 40% after 3% or 6% PGJ, respectively, was found in comparison to VEGF-treated cells.

### 3.5. Effect of Pomegranate Juice on Angiogenic Genes Expression

To confirm the anti-angiogenic properties of PGJ, we analyzed the expression of mRNA angiogenic genes in HBMEC cells after treatment with VEGF-A alone or in combination with 3% and 6% PGJ. Compared to cells exposed to VEGF-A alone, we observed that several angiogenic genes were downregulated in cells treated with VEGF-A in combination with PGJ (Table 2).

### 3.6. Combination of Pomegranate Juice with Proteosome Inhibitor Bortezomib

**Figure 5:**
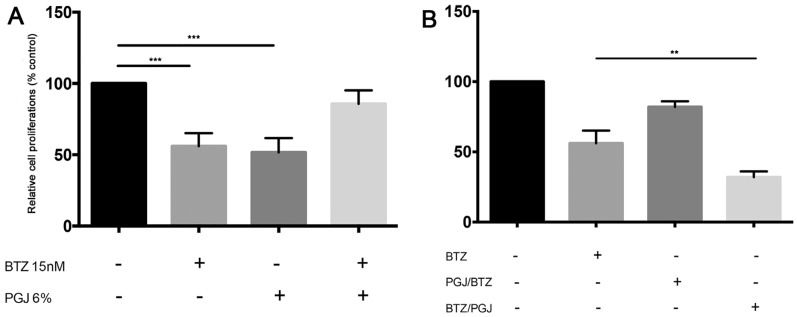
(**A**) the survival assay in U266 cell line treated with Bortezomib (BTZ) alone and in combination with PGJ. *Bars* represent the mean ± SEM of four independent experiments. *** *p* < 0.0001 versus untreated cells; and (**B**) the survival assay in U266 cell line treated alternating BTZ/PGJ or PGJ/BTZ combinations for treatment of 24 h each one. *Bars* represent the mean ± SEM of four independent experiments. * *p* < 0.05; ** *p* < 0.001; *** *p* < 0.0001.

For its anti-proliferative and anti-angiogenic properties, we tested in vitro the combination of PGJ with the proteasome inhibitor BTZ, a first-line drug used for MM therapy. We observed that combination of PGJ (6%) with BTZ inhibited the cytotoxicity of the drug in MM cells (Figure 5A). It may be linked to the G0/G1 cell cycle arrest induced by PGJ that could protect MM cells from BTZ cytotoxic effects. For this reason, we used alternating BTZ/PGJ or PGJ/BTZ combinations for treatment of 24 h each one. Before the addition of the second compound, cells were washed and resuspended in drug-free medium. Pre-treatment with PGJ inhibited cytotoxic effect of BTZ (Figure 5B), probably for the same mechanism that led to the failure of concurrent combination PGJ-BTZ. On the contrary, treatment with PGJ after BTZ improved the cytotoxic effect in MM cell lines (*p* < 0.0001).

## 4. Discussion

It is well-known that *Punica granatum* extracts contain bioactive compounds with anti-cancer actions leading to their use in a number of randomized clinical trials for prostate cancer [39]. Accumulated experimental evidence demonstrates that PGJ inhibits tumor proliferations [40] and induces apoptosis through a nuclear factor-kB-dependent mechanism in vitro and in mice [41]. Previous studies in vitro have also shown that pomegranate metabolites were able to inhibit prostate cancer cell proliferation [42]. The treatment with PGJ showed anticancer effects also in some cancer lines of colon (HT29 and HCT116), liver (HepG2 and Huh7), and breast (MCF-7 and MDA-MB-231) [43]. Our data demonstrated the anti-proliferative potential of PGJ in MM cells and its ability to improve the cytotoxic effect of the proteosome inhibitor BTZ. PGJ contains a number of potential active compounds including organic acids, vitamins, sugars, and phenolic components that are all responsible of the pro-apoptotic effects of PGJ observed in tumor cell lines. The phenolic components include phenolic acids: principally, hydroxybenzoic acids (such as gallic acid and ellagic acid) [44], hydroxycinnamic acids (such as caffeic acid and chlorogenic acid) [45], anthocyanins, including glycosylated forms of cyanidin, delphinidin, and pelargonidin [46], gallotannins and ellagitannins [44]. However, the concentration and the contents of these compounds vary due to growing region, climate, cultivation practice, and storage conditions [45,47]. In particular, it has been reported that the anthocyanins, which are major components of PGJ, were found to have binding affinity against eicosanoid receptors (e.g., peroxisome proliferator-activated receptors α and γ), by this way regulating gene expression and suppressing chemically induced carcinogenesis [48]. Several data demonstrated that the pomegranate leaves, stem and flower extracts modulate cell cycle progression and induce apoptosis in human MM cells through G2/M and S phase cell cycle arrest and mitochondrial membrane permeabilization [49]. It has also been reported that pomegranate induces apoptosis in leukemia cell lines and its polyphenols are responsible for these pro-apoptotic properties [50]. Our results confirm the anti-proliferative effect of PGJ in MM cell lines with a block of cell cycle in G0/G1 phase. Moreover, it upregulated PPARγ mRNA expression, which has been reported to induce apoptosis in MM cells [51,52] and be involved in the induction of G0/G1 phase cell cycle arrest [53]. In addition, accumulated evidence demonstrated that PGJ inhibits tumor angiogenesis and cell invasion [40] Angiogenesis is a constant hallmark of MM progression with prognostic potential [38,54]. In the bone marrow, increased vascularization correlated with a poor prognosis for MM patient [38]. Moreover, it has been reported that MM tumor progression is dependent on endothelial progenitor cells-trafficking (*targeting vasculogenesis to prevent progression in multiple myeloma*). For this reason, we investigated the effect of PGJ on angiogenesis and cell migration/invasion. Interestingly, we observed an inhibitory effect on the tube formation, microvessel outgrowth aorting ring and decreased cell migration and invasion as showed by wound-healing and transwell assays, respectively. Analysis of angiogenic genes expression in endothelial cells confirmed the anti-angiogenic properties of pomegranate. Therefore, PGJ administration could represent a good tool in order to identify novel therapeutic strategies for MM treatment, exploiting its anti-proliferative and anti-angiogenic effects.

Furthermore, we assessed the effect of PGJ treatment in combination with the proteosome inhibitor BTZ. Our results demonstrated that the concurrent administration of PGJ-BTZ on MM cells in vitro reduced the cytotoxic effect of proteosome inhibitor. We hypothesized that block of cell cycle in GO/G1 induced by PGJ could protect MM cells from bortezomib activity. We therefore evaluated sequential exposure of MM cells to the two compounds and found that the sequence of PGJ followed by BTZ did not add any cytotoxic effect to BTZ alone, thus confirming our hypothesis. On the contrary, by exposing MM cells to BTZ followed by PGJ, we observed an increase cytotoxic effect.

This is a pre-clinical model of potential benefits of bortezomib-PGJ combination. We propose using the entire pomegranate juice as a nutraceutical means, able to induce a modulation of the well-known pharmacological function of bortezomib in multiple myeloma, due to the metabolic relationships of individual components present in the whole pomegranate juice and not to a single one. Indeed, several previous studies have well shown that the single phytochemical agents are not able to induce the same effects of the entire pomegranate juice [55,56].

A phase 1 study has been planned to verify potential side effects in patients undergoing treatment, since the effect of PGJ on CYP450 or biochemical interactions is unknown. Most recently, it has been reported that the proteasome-inhibitory and anticancer activity of bortezomib can be blocked by green tea polyphenols, quercetin, myricetin and ascorbic acid through their interaction with the structure of a boronic acid to form boronic ester complexes [57,58]. It is worth noting that the drug has been administered in combination with a variety of antitumor agents in patients with cancer without significant alterations to its pharmacokinetic or pharmacodynamic profile (as reviewed by [4]).

## 5. Conclusions

In conclusion, the current work supports the evidence that PGJ could play a key role of a future nutraceutical approach for treatment of multiple myeloma in order to optimize the pharmacological effect of BTZ, especially as an adjuvant after treatment. Other stimulating studies are necessary in this field in order to better clarify all of the biochemical mechanisms that supervise these interesting antiproliferative and antiangiogenic effects. Future and stimulating studies of PGJ in combination with BTZ or other anti-MM agents are warranted.

## Figures and Tables

**Table 1:** Phenolic compounds composition (mg·L^−1^) of pomegranate juice (Wonderful variety).

Compound	Concentration (mg·L^−1^)
gallic acid	18.2
ellagic acid	97.5
ellagic acid glucoside	11.7
α-punicalagin	3.1
β-punicalagin	6.5
delphinidin 3,5-diglucoside	110.5
cyanidin 3,5-diglucoside	242.8
pelargonidin 3,5-diglucoside	9.3
delphinidin 3-diglucoside	60.4
cyanidin 3-diglucoside	180.6
pelargonidin 3-diglucoside	12.1

**Table 2:** Effect of 6% PGJ on the expression levels of angiogenic genes in HBMEC. Reported data are expressed by relative quantification (fold change) using a 2^−ΔΔCt^ method. VEGF-A treated cells was of the control.

Genes	VEGF	PGJ + VEGF	*p* Value
VEGF	43	0.036	*p* < 0.001
ADAMST1	23	0.041	*p* < 0.001
CXCL12	16	0.136	*p* < 0.001
CXCL2	9	0.038	*p* < 0.001
FGF2	32	0.011	*p* < 0.001
FIGF	12	0.065	*p* < 0.05
IL12A	9	0.030	*p* < 0.001
IL8	21	0.007	*p* < 0.01
MMP2	5	0.031	*p* < 0.001
PDGFB	15	0.116	*p* < 0.001
VEGFB	19	0.028	*p* < 0.001
VEGFC	18	0.008	*p* < 0.001
